# Predicting Running Vertical Ground Reaction Forces Using Neural Network Models Based on an IMU Sensor

**DOI:** 10.3390/s25133870

**Published:** 2025-06-21

**Authors:** Shangxiao Li, Jiahui Pan, Dongmei Wang, Shufang Yuan, Jin Yang, Weiya Hao

**Affiliations:** 1 Research Center for Sports Psychology and Biomechanics, China Institute of Sport Science, Beijing 100061, China; lishangxiao@ciss.cn (S.L.);; 2 College of Human Sport Science, Beijing Sport University, Beijing 100084, China; 3 College of Human Sport Science, Shanghai University of Sport, Shanghai 200438, China

**Keywords:** artificial neural network, wearable sensor, running-related injuries, synchronization algorithm

## Abstract

Vertical ground reaction force (vGRF) plays an important role in the study of running-related injuries (RRIs). This study explores the synchronization method between inertial measurement unit (IMU) and vGRF data of running and develops ANN models to accurately predict vGRF. Fifteen runners participated in this study. Acceleration data and vGRF values of eight rearfoot strikers and seven forefoot strikers running at 12, 14, and 16 km/h were collected by a single IMU and an instrumented treadmill. The sliding time window synchronization (STWS) algorithm was developed to sync IMU data with vGRF data. The wavelet neural network model (WNN) and feed-forward neural network model (FFNN) were adapted to predict vGRF using three-axis or sagittal-axis acceleration data in the stance phase, respectively. One rearfoot striker and one forefoot striker were randomly selected as a test set, while the other participants formed training sets. After synchronization, mean absolute errors for stride time of the IMU and vGRF data were less than 11.2 ms. The coefficient of multiple correlations for vGRF measured curves and predicted curves was more than 0.97. The normalized root mean square errors (NRMSEs) between two curves were 4.6~9.2%, and R^2^ was 0.93~0.99. For peak vGRF, the NRMSEs were 1.6~8.2%, except for rearfoot strike runners at 16 km/h using the FFNN model (10.7% and 11.1%). The Bland–Altman plots indicate that the errors for both the WNN and FFNN models are within acceptable limits. The STWS algorithm can effectively achieve the data synchronization between the IMU and the force plate during running. Both WNN and FFNN models demonstrated good accuracy and agreement in predicting vGRF. Using sagittal-axis acceleration data may be an ideal model with good prediction accuracy and less input data. This work provides direction for developing ANN models of personalized monitoring of lower limb load.

## 1. Introduction

Running is a popular form of physical activity due to its affordability and easy accessibility. While running has a positive effect on health and may potentially increase longevity [1,2], it may also result in running-related injury (RRI) [3,4]. Most RRIs can be categorized as “overuse” injuries, and biomechanical factors may be related to the development of RRIs [5,6,7].

Ground reaction force (GRF), particularly the peak vertical ground reaction force (vGRF), loading rate, and impulse, as well as other factors, are important biomechanical factors during running [8]. It was revealed that an increased vertical loading rate is associated with greater RRI [5,9], specifically with patellofemoral pain, tibial bone stress injury, plantar fasciitis, etc. [10]. Moreover, reducing loading rates may help in the prevention of stress fractures [8]. Long-term monitoring of the GRF, specifically loading rate during outdoor running, may facilitate the early detection of the risk of RRI. However, measuring loading rate requires accurate vGRF measurement, which generally requires a force plate and is usually only possible in a laboratory. Force plates are not always available, and direct vGRF measurement is difficult outside the lab. Instrumented insoles may be used to measure vGRF, but they have shortcomings such as insufficient accuracy, limitations imposed by equipment and usage conditions, complex data processing, and poor long-term stability. Developing portable methods to acquire vGRF without force plates is crucial for measuring loading rates outside the lab, which is eagerly needed.

Artificial neural network (ANN) is a computational model mimicking human brain neural networks. ANN has powerful learning algorithms that form models based on empirical data and then predict outcome values when fed with new data. Compared with traditional mathematical methods, ANNs offer greater advantages in handling complex motion data, improving prediction accuracy, and enhancing adaptability. Moreover, ANNs can be used for various applications, including effective vGRF prediction [11,12]. One study estimated running vGRF using a feed-forward neural network (FFNN) model and a uniaxial accelerometer. It achieved an average root mean square error (RMSE) of less than 0.017 BW and a cross-correlation coefficient greater than 0.99 compared to instrumented treadmill data [11]. Wang et al. predicted vGRF using a wavelet neural network (WNN) model and kinematic variables, achieving a normalized root mean square error (NRMSE) of peak force less than 7.5% [12]. It indicates that both FFNN and WNN are capable of predicting vGRF, as they are common ANN models that use sigmoid and wavelet functions as activation functions, respectively.

Wearable sensors have been viewed as one of the plausible alternatives to capture human motion in an unconstrained environment, especially during running. Inertial measurement units (IMUs) are among the most widely used and cost-effective wearable sensors [13,14]. Therefore, predicting biomechanical variables with IMUs using ANN models may be used to daily monitor biomechanical RRI risk factors without expensive force-measuring equipment. One key issue associated with establishing reliable prediction IMU-based models is the accurate and effective synchronization of IMU with other measuring devices to establish a one-to-one correspondence between the IMU data and other measuring devices. This precise correspondence ensures the reliability and effectiveness of the trained neural network model to predict vGRF through machine learning. However, it is hard to synchronize the IMU with other measuring devices, and synchronizing through a third-party device such as a mobile phone may have data offset. Some studies used multiple jumps performed before and/or after running to synchronize IMUs with the treadmill using cross-correlation analysis [15,16,17]. However, this required extra jump tests, thereby increasing the workload for participants and testers. Comparing the data curves to calculate the minimum error [18] may be an effective method to find synchronization points, thereby allowing the synchronization of IMU with other measuring devices. Synchronization is achieved during data processing, without the need for additional testing.

The purpose of this study was to predict running vGRF using ANN models based on IMU data. The first aspect of this study aims at developing an algorithm to synchronize IMU and vGRF data. The second purpose of this study is to explore the predicting accuracy of different data types (three-axis or sagittal-axis acceleration data) and different ANN models (FFNN or WNN).

## 2. Methods

### 2.1. Participants

In this study, 15 high-level runners (8 males and 7 females) were recruited. Age, height, and weight for the participants were 21.2 ± 2.2 years, 169.2 ± 6.7 cm, and 59.2 ± 7.6 kg, respectively. Participants had 6.4 ± 2.3 years of running experience and ran 72.4 ± 22.2 km weekly. Inclusion criteria were as follows: (1) >3 years of running experience, >30 km/week; (2) generally healthy; and (3) experience running on a treadmill. A previous study on usability engineering suggested that 5 participants are already adequate, and not much additional value will be obtained beyond 10 [19]. Therefore, in this study, 8 rearfoot strikers and 7 forefoot strikers were recruited. This study was conducted according to the guidelines of the Declaration of Helsinki and approved by the Ethics Committee of the China Institute of Sport Science (approval number: CISSLA-2021002), and informed consent was obtained from all subjects involved in this study.

### 2.2. Data Collection

An IMU (Lpms-B2, ALUBI, Guangzhou, China) was fixed with a strap at the left shank 8 cm above the medial malleolus, with its vertical axis oriented upward. The collection frequency of the IMU built-in accelerometer was 200 Hz. A standing calibration trial was performed in anatomical position, and the IMU internal sensor was calibrated using the Lpms Control software, version 1.3.5.

The vGRF value was recorded using an instrumented treadmill (h/p/cosmos Gaitway II, 250 Hz, Traunstein, Germany) equipped with two Kistler force platforms (Kistler Instrumente AG, Winterthur, Switzerland) and Gaitway 2.0 software (h/p/cosmos Gaitway II, 250 Hz, Traunstein, Germany). Two testers clicked “Start” in Gaitway 2.0 and Lpms Control software to begin data collection. After a 5-minute treadmill warm-up at self-selected speed, participants ran at 12, 14, and 16 km/h for 30 s each, with 2–3 min rest between trials. The speed chosen in this study was their daily running pace.

### 2.3. Data Processing

The vGRF values underwent a low-pass filtering using a Gaussian impulse response with a Laplace operator, automatically performed by the software of the h/p/cosmos treadmill software [20], and were resampled to 200 Hz, with the same frequency as the IMU data.

A gait cycle (stride) was defined by consecutive left toe-offs. For IMU data, toe-off was marked by the minimum coronal angular velocity before peak velocity [21]. For vGRF data, toe-off was when vGRF dropped below 50 N [22]. The custom program was developed in Python 3.7.

The experimental collection in this study only ensures that the two different types of data are roughly synchronized. Due to the time delay caused by software collection and the reaction time of the testing personnel, the two groups of data are not exactly synchronized. The synchronization of IMU and vGRF data was based on gait’s periodic nature, despite variability in parameters like stride time, length, and width across cycles [23]. Thus, consecutive gait cycles in different signals (e.g., IMU and vGRF) must have a synchronization point. After synchronization, stride times in both signals should correspond one by one, minimizing relative errors. Therefore, we developed the sliding time window synchronization (STWS) algorithm.

### 2.4. Data Synchronization

**Figure 1:**
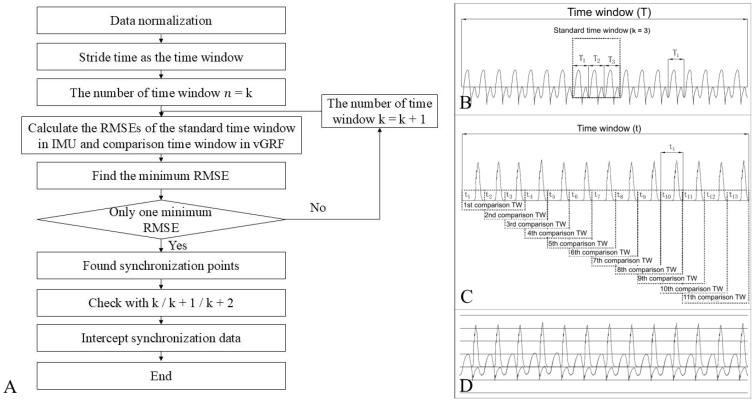
Illustration of sliding time window synchronization algorithm. (**A**) Flow diagram of sliding time window synchronization algorithm. (**B**) Standard time window at IMU data. (**C**) Comparison time window group at vGRF data (TW represents “time window”). (**D**) Case diagram of synchronous data after sliding time window synchronization.

The STWS algorithm (Figure 1) used IMU data (three-axis or sagittal-axis acceleration) and vGRF data, both divided into continuous subcurves (gait cycles) with specific stride times. IMU subcurves are defined as time window T, and vGRF subcurves as time window t. A standard time window is created from k consecutive strides in IMU data, using the intermediate gait period to avoid synchronization issues. For vGRF data, k consecutive strides form a comparison time window group, starting from the first stride. The RMSE of stride times is calculated by comparing the standard time window of IMU data with each comparison time window of vGRF data, starting with $k_{0}$ = 3.

The synchronization points between IMU and vGRF data were determined by the minimum RMSE. If multiple minimum errors occurred, the comparison time window group’s stride number was increased (*k* = *k* + 1) until the minimum error was found. After identifying the synchronization point, *k* + 1 and *k* + 2 were used to confirm the computation. This point was then used to align IMU and vGRF data, ensuring all strides matched (Figure 1).

A total of 52 consecutive running trials were conducted in this study. There are 30 trials when synchronized with *k* = 3, 17 trials when synchronized with *k* = 4, 4 trials when synchronized with *k* = 5, and 1 trial when synchronized with *k* = 6. Moreover, the STWS algorithm, verified with synchronized infrared motion capture and force treadmill vGRF data during running (similar to [12]), correctly identified synchronization points in all 12 trials.

After synchronization, 12–28 gait cycles were obtained at each speed for each participant. Foot strike was defined as the moment when the vertical ground reaction force (vGRF) exceeded 50 N. vGRF was normalized by body weight (BW), and IMU and vGRF data during the stance phase were interpolated to 101 points using spline interpolation to represent 0–100% of the stance phase. Foot strike patterns were analyzed via foot strike angle from videos [24]. After excluding 11 incomplete or abnormal cycles, rearfoot strike (RFS) data included 389 cycles from 8 participants (3 males, 5 females), with one participant (36 cycles) as a test set and the rest (353 cycles) as training sets. Forefoot strike (FFS) data included 423 cycles from 7 participants (5 males, 2 females), with one participant (59 cycles) as a test set and the rest (364 cycles) as training sets. We have ensured that the training and test datasets are derived from different individuals to prevent the model from merely learning the gait patterns of specific participants.

### 2.5. WNN and FFNN Model Construction

**Figure 2:**
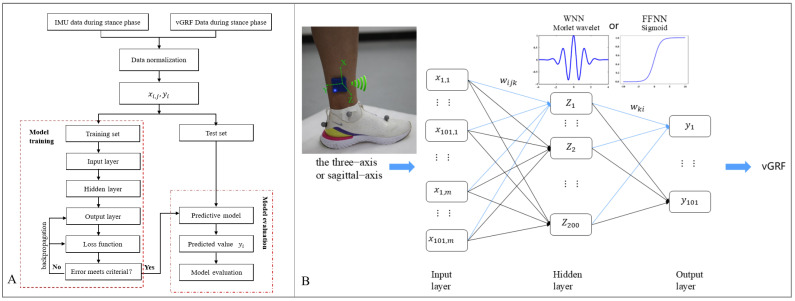
Flow charts of WNN and FFNN models. (**A**) Model training and prediction evaluation. (**B**) The input selection and model structure.

Both WNN and FFNN are fully connected neural networks, including an input layer, varying numbers of hidden layers, and an output layer (Figure 2). The main difference between the two models is that the hidden layer activation function is different (Figure 2).

The input layer was composed of {$x_{1,1}$, …, $x_{101,1}$, ……, $x_{1,m}$, …, $x_{101,m}$}, with *m* = 3 or *m* = 1, indicating that three-axis acceleration data or sagittal-axis acceleration IMU data. This study used the optimized WNN and FFNN model (Figure 2), taking acceleration and label data of each stride, totaling 101 data points, as a sample input so that the model could fully identify the information and find accurate rules.

For the WNN model, the activation function was the Morlet wavelet ψ(x):

(1) $$\psi \left(x\right)=exp\left(-\frac{x^{2}}{2}\right)\cos\left(5x\right)$$

The output of the hidden layer is as follows:

(2) $$z_{k}=\psi \left(\frac{\sum_{i=1}^{n}\sum_{j=1}^{m}w_{ijk}x_{ij}-b_{k}}{a_{k}}\right)$$

where $w_{ijk}$ (i = 1, 2, …, 101, j = 1, 2, …, m) is the connection weight of the hidden layer, $b_{k}$ is the shifting factor, and $a_{k}$ is the scaling factor.

The predicted result of the output layer is as follows:

(3) $$y_{i}=\sum_{k=1}^{K}w_{ki}z_{k}-b_{i}$$

where $y_{i}$ is the predicted value, *K* is the node number of the hidden layer, $w_{ki}$ (k = 1, 2, …, *K*) is the connection weight of the output layer, and $b_{i}$ represents bias terms.

For the FFNN model, the activation function was a sigmoid function $S\left(x\right)$:

(4) $$S\left(x\right)=\frac{1}{1+e^{-x}}$$

The output of the hidden layer is as follows:

(5) $$z_{k}=S\left(\frac{\sum_{i=1}^{n}\sum_{j=1}^{m}w_{ijk}x_{ij}-b_{k}}{a_{k}}\right)$$

The predicted result of the output layer is the same as in the WNN model.

### 2.6. Model Training

The TensorFlow toolbox 2.11.x was used to build the three-layer WNN and FFNN model using Python 3.7, and the RFS and FFS datasets were trained separately.

The training set was constructed by three-axis acceleration or sagittal-axis acceleration of the IMU and the vGRF data during the running stance phase. To avoid overfitting, the complexity of the model was reduced, and the number of parameters was also minimized. Moreover, $L_{2}$ norm regularization was added to alleviate overfitting. The loss function of the model in this study was expressed as follows:

(6) $$E=\frac{1}{n}\sum_{i=1}^{n}{\left({\hat{y}}_{i}-y_{i}\right)}^{2}+\lambda {\left\|w\right\|}_{2}$$

where *n* is the number of training set samples for RFS or FFS; ${\hat{y}}_{i}$ is the measured value for the sample i; λ = 0.01; and w contains $w_{ijk},\;w_{ki},\;b_{k},\;a_{k},\;and\;b_{i}$.

The Adam optimizer was used, and backpropagation (BP) based on gradient descent and chain derivation rule was used to find the optimal solution of parametric that minimizes the loss function. In this study, hyperparameters such as the number of hidden layer nodes, batch size, and epochs were manually adjusted based on current common strategies for hyperparameter tuning [25]. For the model, hyperparameters included hidden layers (1, 2, and 3), nodes per layer (50, 100, 150, and 200), regularization (*L*1, *L*2), and activation functions (Morlet wavelet and sigmoid). For training, hyperparameters included iterations (50, 100, 150, and 200), batch size (64, 128, and 256), and learning rate (5 × 10^−^³, 5 × 10^−4^, and 5 × 10^−5^). The final model has one hidden layer with 200 nodes; either activation function is acceptable, the batch size is 128, the iterations are 100, and the learning rate is 5 × 10^−4^. The processor used in this study is an Intel(R) Core(TM) i7-10710U CPU@ 1.10 GHz (Intel, Santa Clara, CA, USA), with a maximum boost frequency of 1.61 GHz and 16.0 GB of RAM.

The model automatically terminated after reaching the specified number of iterations. If the mean squared error (MSE) of the loss function decreased then increased, the hyperparameters, such as iteration number, were adjusted. Once MSE was minimized, the model structure and parameters were saved.

### 2.7. Statistical Analysis

To verify synchronized data accuracy, the mean absolute errors (MAE), maximum absolute errors (MXAE), MRE, and maximum relative error (MXRE) of stride time were calculated for IMU and vGRF data.

The RFS and FFS test sets evaluated the WNN and FFNN models’ prediction results after training. To assess WNN and FFNN accuracy, the coefficient of multiple correlation (CMC), RMSE, NRMSE, and mean absolute percentage error (MAPE) were calculated for measured and predicted vGRF values during the stance phase. The Bland–Altman was calculated using the 95% limits of agreement and mean error between the measured and predicted vGRF peak values. All metrics were expressed as means ± SD.

## 3. Results

The results of this study showed that MAEs of the time parameters of IMU and vGRF data were less than 11.2 ms, and MREs were no more than 1.8%; MXAEs were no more than 55 ms, and MXREs were less than 8.0% (Table 1).

**Figure 3:**
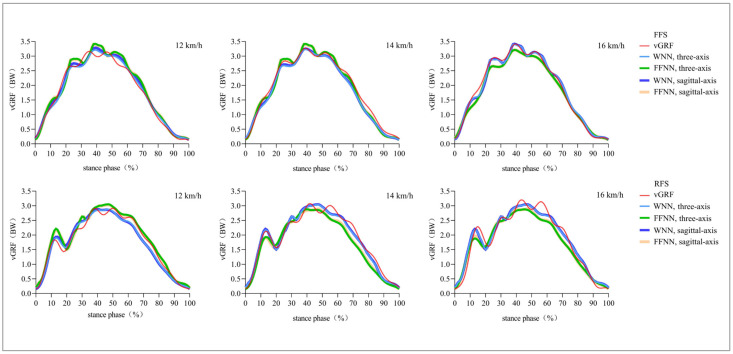
Comparisons of vGRF curves between measured and predicted by WNN and FFNN models using three-axis or sagittal-axis acceleration.

All of the predicted curves by the WNN model or FFNN model were very similar to the measured curve (Figure 3). For FFS runners, CMCs for vGRF predicted and measured curves were more than 0.99 (WNN) and 0.98 (FFNN). NRMSEs between two curves were averaged 4.6~6.3% and 5.7~7.0% (Table 2). For peak vGRF, NRMSEs were averaged to 1.6~3.9% and 4.0~7.9%, and MAPEs were 1.4~3.4% and 3.1~7.7%, respectively (Table 3).

For RFS runners, CMCs of vGRF predicted and measured curves were more than 0.99 (WNN) and 0.97 (FFNN). NRMSEs between two curves were 5.6~6.8% and 6.5~9.2% (Table 2). For peak vGRF, NRMSEs were 2.1~6.3% and 3.3~11.1%, and MAPEs were 1.8~6.0% and 3.1~11.0%, respectively (Table 3).

**Figure 4:**
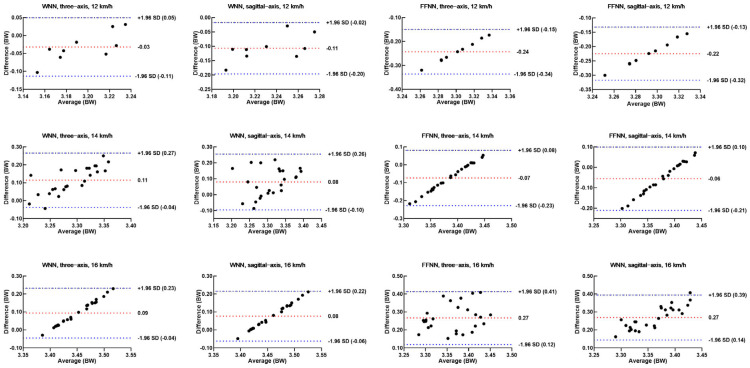
The Bland–Altman analysis for peak vGRF predictions for FFS runners by WNN and FFNN models using three-axis or sagittal-axis acceleration.

The Bland–Altman method was used to analyze the consistency of peak vGRF of the predicted data of WNN for FFS runners. The results showed that errors of peak vGRF were all within the 95% confidence interval, with an average error of 0.11 BW and a maximum error of 0.18 BW. For the predicted data of FFNN using three-axis acceleration, the errors of peak vGRF were all within the 95% confidence interval, with an average error of 0.22 BW and a maximum error of 0.25 BW. For the predicted data of FFNN using sagittal-axis acceleration, only 3.85% (1/26) of peak vGRF at 16 km/h were outside the 95% confidence interval. The errors of peak vGRF at 12 km/h and 14 km/h were all within the 95% confidence interval, with an average error of 0.20 BW and a maximum error of 0.24 BW (Figure 4).

**Figure 5:**
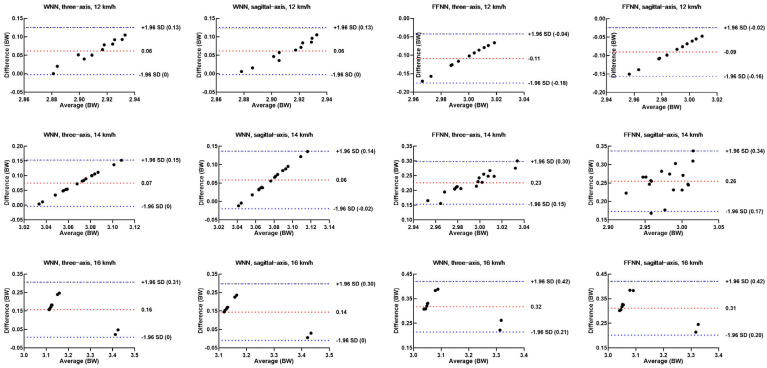
The Bland–Altman analysis for peak vGRF predictions for RFS runners by WNN and FFNN models using three-axis or sagittal-axis acceleration.

For the predicted data of WNN for RFS runners, Bland–Altman results showed that errors of peak vGRF were all within the 95% confidence interval, with an average error of 0.11 BW and a maximum error of 0.19 BW. For the predicted data of FFNN using three-axis acceleration, only 5.56% (1/18) of peak vGRF at 14 km/h were outside the 95% confidence interval. Additionally, the errors of peak vGRF at 12 km/h and 16 km/h were all within the 95% confidence interval, with an average error of 0.24 BW and a maximum error of 0.36 BW. For the predicted data of FFNN using sagittal-axis acceleration, 11.1% (2/18) of peak vGRF at 14 km/h were outside the 95% confidence interval. The errors of peak vGRF at 12 km/h and 16 km/h were all within the 95% confidence interval, with an average error of 0.22 BW and a maximum error of 0.34 BW (Figure 5).

Table 4 compared the prediction results of running vGRF based on IMU. Our models (WNN and FFNN) using single-IMU data (three-axis or sagittal-axis) showed consistent RMSE with previous studies and relatively lower NRMSE values at corresponding speeds.

## 4. Discussion

The STWS results of this study showed that the MAEs were less than 11.2 ms and the MREs were less than 1.8%. MXAEs between stride time of IMU and vGRF data were no more than 55 ms, and MREs were less than 8.0%. The IMU and vGRF data are considered properly synchronized, and the STWS algorithm can effectively achieve the synchronization between the IMU and the force plate. Furthermore, synchronized data validated the STWS algorithm, correctly identifying synchronization points in all 12 trials and further verifying its accuracy.

This study compared joint kinematics from an inertial sensor system and an optical motion capture (OMC) system. The OMC joint angle curve was vertically shifted over the inertial sensor curve using an optimization algorithm to minimize the sum of squared errors, achieving synchronization [18]. Notably, this study used 16 IMUs and compared the same joint angle of only one gait cycle [18]. In our study, we use a sliding time window method to calculate the minimum RMSE of multiple continuous gait cycles, with only one single IMU used to achieve synchronization. The STWS algorithm used in our study can overcome the application limitations of IMU and provide a novel idea to solve the synchronization problem between IMU and other measurement systems.

The vGRF sustained by lower limbs may be the key monitoring index for injury prevention in running [8,9]. However, it is difficult to obtain real-time data feedback for injury risk assessment in daily running. An ANN model using a wearable sensor overcomes limitations of conventional methods and offers effective monitoring for outdoor running, injury prevention, and rehabilitation. This study developed two vGRF ANN models (WNN and FFNN) using single-IMU data (three-axis or sagittal-axis acceleration) from the medial malleolus. FFS was associated with a more plantarflexed ankle at initial contact, a smaller peak ankle dorsiflexion angle, and greater ankle excursion compared with RFS [28]. Running with RFS imposed higher biomechanical loads on overall ground impact and knee and patellofemoral joints, whereas FFS imposed higher biomechanical loads on the ankle joint and Achilles tendon [28]. RFS experiences a higher loading rate, which may lead to an increased risk of impact-related injuries to the lower extremity [29]. Therefore, FFS and RFS were studied separately due to the differences in kinematic and kinetic characteristics between these foot-strike patterns during running [29]. Results of this study showed that for three-axis data, the CMCs of WNN and FFNN between curves were no less than 0.95, and NRMSEs were less than 9.2%. All NRMSEs of peak vGRF were no more than 8.2%, except for the FFNN model of RFS at 16 km/h (NRMSE = 11.1%). For sagittal-axis data, the CMCs between curves were higher than 0.97, and NRMSEs were less than 8.8%. All NRMSEs of peak vGRF were no more than 7.3%, except for the FFNN model of RFS at 16 km/h (NRMSE = 10.7%). Our data indicate that both FFNN and WNN models based on three-axis or sagittal-axis acceleration data of IMU can successfully predict vGRF with very few errors and with good agreement between predicted and measured data. This error may have a relatively small impact on the calculation of the loading rate. Therefore, it may have a relatively good effect on predicting RRIs in the later stage.

The vGRF estimation errors from ANN models using IMU data in this study were comparable to previous studies at corresponding speeds [15,26]. One study used three machine learning models with a sacral IMU to estimate peak vGRF at 9, 11, and 13 km/h, achieving RMSEs of 0.13 BW and MAPEs of 3.8% [26]. Another study used a sacral biaxial accelerometer to predict GRF waveforms during uphill and downhill running at 9, 12, and 15 km/h, with RMSEs of 0.16 BW and NRMSEs of 6.4% [30]. Ngoh’s study showed RMSEs of 0.015–0.017 BW, but the running speed was quite slow, at 8, 9, and 10 km/h [11]. In addition, the accuracy of the prediction model may be affected by IMU placement. Placing the IMU on the foot captures GRF changes directly but may yield unstable data due to complex joint movements. The thigh and sacrum provide movement data but do not directly reflect lower leg and ankle characteristics, potentially affecting prediction. The shank is relatively flat and has fewer muscles, providing a stable platform for the IMU and reducing data errors from muscle tremors or skin folds. Therefore, this study positioned the IMU on the shank, and the models showed good accuracy. Notably, parts of the Bland–Altman plots showed linear trends, which indicate that the error between vGRF and predicting value is positively related to the vGRF magnitude. This issue may be influenced by the fact that while vGRF was normalized to body weight, the IMU data were not normalized. This could influence the training process and lead to the suppression of other informative features. Furthermore, the sample size of this study is relatively small. Although there is a large amount of gait cycle data, it originates from only 7 rearfoot strikers and 6 forefoot strikers. Additionally, the running speed is fixed, so the corresponding vGRF is also relatively concentrated. This limited number of participants and fixed speed may result in a relatively concentrated distribution of the training data, which could prevent the model from effectively capturing the distribution characteristics of the data [31]. Moreover, each test set comprises data from just one participant, and the sample size of gait cycles is relatively small. Previous studies have suggested using at least 40 samples for Bland–Altman analysis [32]. Future research should aim to include a larger number of samples and a wider range of running speeds for both training and test sets to improve the robustness and generalizability of the model. However, overall, the Bland–Altman plots indicate that the errors for both the WNN and FFNN models are within acceptable limits.

FFNNs are general-purpose neural networks that are highly versatile and can be applied to a wide range of tasks. WNNs combine wavelets with localization properties and self-learning neural networks, which is beneficial for general approximation ability. They have proved to be powerful tools for analyzing time-series features and simulating nonlinear characteristics [33]. In our study, the RMSEs and MAPEs of peak vGRF based on WNN for 12 and 14 km/h were less than 0.13 BW and 3.28%, similar to the previous study [26], and the RMSEs and MAPEs of FFNN were slightly higher, with 0.26 BW and 7.9%. Overall, both WNN and FFNN accurately predicted vGRF for RFS and FFS. There was little difference between three-axis and sagittal-axis IMU data for prediction. Using uniaxial accelerometers can simplify the estimation of vGRF and reduce the amount of computation and may be more useful in predicting vGRF. This study suggests improving vGRF monitoring systems with convenient sagittal-axis accelerometers and informs the development of accelerometer-integrated insoles or socks to prevent running injuries.

There are limitations in this study. Firstly, the models were established based on data from a small number of subjects and a relatively small number of gait cycles. Age, leg length, and running strategy may affect vGRF. Different populations should be included in future research. Furthermore, the results would be more comprehensive with leave-one-subject-out or k-fold cross-validation. Thirdly, the running speeds tested were 12, 14, and 16 km/h with a single IMU on the left leg. Future work should explore other speeds and IMU placements. Our data were collected from the treadmill. We speculate that if sufficient ground-running data are accumulated in the future, a model with better performance can be trained. Moreover, this study only predicted the vGRF. In fact, the GRF in AP (anteroposterior) and ML (mediolateral) axes during running is also very important. Meanwhile, assessing the loading conditions of the lower limb bones solely through vGRF may have certain limitations. Future research could consider incorporating the load generated by muscle contractions into the analysis to gain a more comprehensive understanding of the force mechanisms of lower extremity bones and their relationship with RRI. This represents a phased achievement of our research and marks a certain degree of substantive progress. Nevertheless, we acknowledge that further training will be necessary for its future real-world applications. Despite limitations, this study developed a synchronization algorithm for IMU and force plate data and is applicable to periodic movement data synchronization. It demonstrated that ANN models based on one IMU can accurately predict running vGRF. Specifically, using sagittal-axis acceleration can monitor vGRF for personalized runners outside the lab, thus offering new technical approaches for some longitudinal RRI prevention by accumulated load computation. The neural network models have high application value, considering the easy acquisition method (portable sensor), less input data (single-axis acceleration), and simple model with good performance.

## 5. Conclusions

This study developed a sliding time window synchronization (STWS) algorithm, which has been demonstrated to be effective in the running data synchronization between IMU and force plate. The STWS algorithm was based on time variability of periodic movements, so it may be used for synchronizing calculation of different types of movements, including running, walking, etc.

Both WNN and FFNN models demonstrated good accuracy and agreement in predicting vGRF. Using sagittal-axis data may be an ideal model with good prediction accuracy and less input data. This work provides a solid direction for developing ANN models of personalized monitoring of lower limb load and may provide further protection from RRIs in runners.

## 6. Patents

This study developed a synchronization algorithm for IMU and force plate data, which is applicable to periodic movement data synchronization and has been patented. The method involves synchronizing and comparing data within specific time windows to enhance the accuracy and reliability of motion analysis. This patented technique (A Method for Kinematic Data Acquisition and Analysis Based on Time-Window Comparison and Synchronization, CN115956902B, 2024) is integral to the methodology presented in the Method Section of this manuscript.

## Figures and Tables

**Table 1:** Differences in stride time between IMU data and vGRF data calculated by the sliding time window synchronization algorithm.

Speed (km/h)	Stride Number	MAE (ms)	MXAE (ms)	MRE (%)	MXRE (%)
12	271	9.8 ± 8.8	45	1.4 ± 1.3	6.8
14	283	11.2 ± 10.6	55	1.8 ± 1.6	8.0
16	260	10.3 ± 10.2	50	1.6 ± 1.6	7.6

MAE, mean absolute error; MXAE, maximum absolute error; MRE, mean relative error; MXRE, maximum relative error.

**Table 2:** Evaluation of prediction results of running vGRF curves by WNN and FFNN models using three-axis or sagittal-axis acceleration.

Model	Strike Pattern	Speed (km/h)	Three-Axis Acceleration				Sagittal-Axis Acceleration			
			CMC	RMSE (BW)	NRMSE (%)	R^2^	CMC	RMSE (BW)	NRMSE (%)	R^2^
WNN	FFS	12	0.99	0.15 ± 0.04	4.92 ± 1.39	0.98 ± 0.01	0.99	0.17 ± 0.04	5.30 ± 1.30	0.98 ± 0.01
		14	0.99	0.21 ± 0.06	6.25 ± 1.91	0.98 ± 0.01	0.99	0.21 ± 0.07	6.21 ± 1.97	0.98 ± 0.01
		16	0.99	0.16 ± 0.05	4.58 ± 1.57	0.98 ± 0.01	0.99	0.16 ± 0.06	4.66 ± 1.65	0.98 ± 0.01
	RFS	12	0.99	0.16 ± 0.04	5.81 ± 1.40	0.96 ± 0.02	0.99	0.19 ± 0.06	6.19 ± 1.97	0.96 ± 0.02
		14	0.99	0.17 ± 0.04	5.65 ± 1.37	0.94 ± 0.03	0.99	0.17 ± 0.04	5.60 ± 1.38	0.95 ± 0.02
		16	0.99	0.21 ± 0.07	6.70 ± 2.39	0.93 ± 0.02	0.99	0.22 ± 0.08	6.79 ± 2.47	0.94 ± 0.02
FFNN	FFS	12	0.98	0.22 ± 0.06	7.03 ± 2.03	0.97 ± 0.01	0.98	0.22 ± 0.06	6.87 ± 1.85	0.97 ± 0.01
		14	0.99	0.20 ± 0.06	5.91 ± 1.71	0.98 ± 0.01	0.99	0.19 ± 0.05	5.70 ± 1.56	0.98 ± 0.01
		16	0.99	0.21 ± 0.08	6.16 ± 2.22	0.99 ± 0.01	0.99	0.20 ± 0.07	5.82 ± 1.96	0.99 ± 0.01
	RFS	12	0.98	0.19 ± 0.11	6.63 ± 3.42	0.97 ± 0.01	0.98	0.19 ± 0.06	6.47 ± 2.09	0.97 ± 0.01
		14	0.97	0.29 ± 0.10	9.21 ± 3.28	0.97 ± 0.01	0.97	0.27 ± 0.09	8.83 ± 3.01	0.97 ± 0.01
		16	0.97	0.29 ± 1.11	8.80 ± 3.46	0.96 ± 0.02	0.97	0.28 ± 0.10	8.67 ± 3.18	0.96 ± 0.02

FFS, forefoot strike; RFS, rearfoot strike; CMC, coefficient of multiple correlations; RMSE, root mean square error; NRMSE, normalized root mean square error; BW, body weight.

**Table 3:** Evaluation of prediction results of peak vGRF of running by WNN and FFNN models using three-axis or sagittal-axis acceleration.

Model	Strike Pattern	Speed (km/h)	Three-Axis Acceleration			Sagittal-Axis Acceleration		
			RMSE (BW)	NRMSE (%)	MAPE (%)	RMSE (BW)	NRMSE (%)	MAPE (%)
WNN	FFS	12	0.05 ± 0.01	1.60 ± 0.10	1.40 ± 0.79	0.12 ± 0.01	3.66 ± 0.29	3.38 ± 1.40
		14	0.12 ± 0.01	3.67 ± 0.37	3.27 ± 1.64	0.13 ± 0.02	3.85 ± 0.50	3.26 ± 2.12
		16	0.08 ± 0.01	2.24 ± 0.22	1.80 ± 1.32	0.07 ± 0.01	2.04 ± 0.20	1.46 ± 1.44
	RFS	12	0.07 ± 0.01	2.39 ± 0.13	2.08 ± 1.04	0.09 ± 0.01	3.17 ± 0.35	2.67 ± 1.65
		14	0.08 ± 0.01	2.52 ± 0.14	2.15 ± 1.13	0.06 ± 0.01	2.11 ± 0.10	1.80 ± 0.96
		16	0.19 ± 0.01	6.25 ± 0.42	6.04 ± 0.94	0.19 ± 0.01	5.87 ± 0.39	5.66 ± 0.94
FFNN	FFS	12	0.25 ± 0.02	7.85 ± 0.70	7.67 ± 1.52	0.23 ± 0.02	7.27 ± 0.64	7.09 ± 1.51
		14	0.15 ± 0.03	4.40 ± 0.88	3.58 ± 2.90	0.13 ± 0.03	4.00 ± 0.79	3.13 ± 2.79
		16	0.25 ± 0.03	7.09 ± 0.91	6.78 ± 2.06	0.24 ± 0.03	6.82 ± 0.79	6.56 ± 1.84
	RFS	12	0.11 ± 0.01	3.92 ± 0.28	3.71 ± 1.14	0.10 ± 0.01	3.32 ± 0.22	3.08 ± 1.13
		14	0.26 ± 0.03	8.15 ± 1.03	7.86 ± 1.84	0.21 ± 0.02	7.07 ± 0.58	6.73 ± 1.39
		16	0.36 ± 0.04	11.14 ± 1.24	11.02 ± 1.35	0.34 ± 0.02	10.72 ± 0.69	10.47 ± 0.88

FFS, forefoot strike; RFS, rearfoot strike; RMSE, root mean square error; NRMSE, normalized root mean square error; MAPE, mean absolute percentage error; BW, body weight.

**Table 4:** Comparison of evaluation of prediction results of running vGRF based on IMU.

Study	Model	Number of IMUs	Placement of IMUs	Running Speed (km/h)	RMSE (BW)	NRMSE (%)
Scheltinga et al. [15]	Direct	Multiple (three)	bilateral feet, tibias, thighs, pelvis, and trunk	10, 12, 14	0.19 ± 0.04	7.3 ± 1.8
	Hybrid				0.18 ± 0.04	6.8 ± 1.7
Patoz et al. [26]	Linear regression	Single	Sacral	9, 11, 13	0.12	-
	Support vector regression				0.13	-
	Two-layer neural network				0.13	-
Ngoh et al. [11]	FFNN	Single	right shoe	8, 9, 10	0.015–0.017	-
Johnson et al. [27]	CNN (CaffeNet)	Multiple (five)	Pelvis, bilateral thigh, and bilateral shank	14.4–18	-	13.93
This study	WNN	Single	Shank (three-axis)	12, 14, 16	0.15–0.21	4.92–6.70
	FFNN				0.19–0.29	5.91–9.21
	WNN	Single	Shank (sagittal-axis)		0.17–0.22	4.66–6.79
	FFNN				0.19–0.28	5.82–8.83

FFNN, feed-forward neural network model; CNN, competing convolutional neural network; WNN, wavelet neural network model.

## Data Availability

The data that support the findings of this study are available on reasonable request from the corresponding author. The data are not publicly available due to privacy or ethical restrictions.
